# Editorial: Seafood: nutrition savior or safety hazard?

**DOI:** 10.3389/fnut.2023.1256358

**Published:** 2023-07-26

**Authors:** Sara Sousa, Maria Luz Maia, Laura Guimarães, Valentina F. Domingues

**Affiliations:** ^1^REQUIMTE/LAQV, Instituto Superior de Engenharia do Porto, Polytechnic of Porto, Porto, Portugal; ^2^Center for Research in Health Technologies and Information Systems, Porto, Portugal; ^3^Centro Interdisciplinar de Investigação Marinha e Ambiental (CIIMAR), Universidade do Porto, Matosinhos, Portugal

**Keywords:** food quality, alternative protein sources, nutritional research, safe food production, marine bioactive peptides

Seafood is consumed all around the world. It is considered a high-quality food and an excellent source of vitamins, minerals, proteins, essential amino acids, and fatty acids (1–3). Seafood consumption have been associated with the reduction of inflammatory diseases, stroke, arthritis, hypertension, attention deficit hyperactivity disorder, and reduced risk of Alzheimer's disease, cardiovascular disease, cancer, and depression (1, 4).

Contamination of coastal areas and oceans, originating from human activities, disturbs marine ecosystems, thus causing seafood to be also a potential source of pollutants such as microplastics, pesticides, synthetic musks, persistent organic pollutants, metals, and pharmaceuticals (3–6). There is also the threat of potential biological hazards such as the production and accumulation of natural toxins and allergens (e.g., tetrodotoxin, histamine, ciguatera), as well as parasites and various infectious pathogens, including bacteria and viruses. The consumption of contaminated seafood may, therefore, represent a major hazard to human health (4). Owing to this, the risk-benefit scale for seafood consumption has always been a discussion issue. Nonetheless, the quality decline of the aquatic environment over the years may have toppled toward the risk portion, with several protection agencies issuing policy statements and/or guidance on this conundrum. Overall, it is vital to monitor the aquatic environment and produce more research about new developments and understanding on the safe production, value, and risks of seafood for human consumption. The articles included in this Research Topic are from experts in the fields of food quality, food safety, risk assessment, health hazard, toxicology, and nutritional research.

Fish is highly consumed around the world, representing 17% of the total animal protein consumed by humans (7). The reducing of production costs while maintaining the flesh quality and flavor of aquaculture fish is a challenge. An alternative is the inclusion of plant protein in fish meals. On this aspect, Li et al. combined molecular biology and metabolomics techniques to thoroughly investigate the molecular mechanisms by which different plant proteins affect the proliferation and degradation of muscle proteins of yellow catfish. The authors found soybean meal, peanut meal, cottonseed meal, sesame meal and corn gluten meal regulated the biosynthesis and degradation of muscle protein by affecting the content of vitamin B6, proline, glutamic acid, and phenylalanine (tyrosine) in muscle. The plant proteins improved the flesh quality and texture, despite significantly reducing the growth performance. The authors observed that inhibition of myocyte proliferation-related genes in cottonseed, sesame and corn gluten groups might additionally be regulated by the increase of glutamic acid and the decrease of tyrosine contents (Li et al.). The work provides a needed theoretical basis for further understanding the mechanism of plant proteins regulating flesh quality.

Still related to alternative protein sources, Alvanou et al. evaluated for the first time the incorporation of larvae of black soldier flies (*Hermetia illucens*) in the feeds of aquaculture crayfish. Crustaceans have long been considered a delicacy. The demand for crustaceans, on the one hand, has been increasing worldwide, which can lead to overexploitation of wild species. On the other hand, the aquaculture farming of these species is costly due to the substantial amount of fishmeal required in the feeds (Alvanou et al.). Insects are high in protein and fat, and an alternative of low environmental footprint. The investigation of Alvanou et al. found that 98 days fishmeal replacements of 50 and 100% in juvenile diets increased crayfish survival but negatively affected its growth performance and feed utilization. The inclusion of the fly larvae in the diet also altered the fatty acid levels and profiles of crayfish. The study shed some light on contradictory results available in the literature for other species.

On another relevant topic and with a different approach, Quaresma et al. studied the protein quality of dried salted cod fish, the 3rd highest consumed fish in the European Union. Due to high demand and capture, Atlantic cod is considered a vulnerable species by the International Union for the Conservation of Nature. In comparison, Pacific cod is captured in lower amounts. Considering this, Quaresma et al. evaluated and classified the nutritional quality in relation to the cod species (*Gadus morhua* and *Gadus macrocephalus*) and harvesting areas (Norway and Iceland Exclusive Economic Zones) using an essential amino acid index. They found the amino acid profiles and the index produced full accurate discrimination of the cod species. Discrimination of the location was above 85%. Furthermore, Norwegian cod protein was classified as of high quality, while that of Iceland cod was of useful quality protein. The same five limiting amino acids were found in both species.

By contrast, Gao et al. approached seafood from the innovative perspective of marine bioactive peptides. They studied the potential of peptides from tilapia skin for the treatment of ulcerative colitis, which is a main type of Inflammatory Bowel Disease. Tilapia peptides display anti-inflammatory, antioxidant, antihypertensive, and immunomodulatory properties amongst others (8). The authors employed cell culture assays (CT-26 and HT-29 cell lines) and an induced mouse model of colitis (Dextran sulfate sodium-induced) to evaluate the effects of the peptides. These peptides seemed to inhibit inflammation and apoptosis directly in the colon epithelium, emerging as a potential alternative treatment for inflammatory bowel disease (Gao et al.). The mechanistic investigation points out a role of such bioactive peptides in the protection against external stimuli, which passes through enhancing the barrier function of the colon epithelium, reducing the release of inflammatory cytokines, and suppressing apoptosis.

In sum, the contributing articles address either the benefits of seafood consumption, potential health usages, or alternatives to improve seafood safety and quality. These bring new information on relevant aspects of the topic, opening questions and challenges for future research related to alternative protein sources, the nutrient sensing signaling pathways, quality evaluation and species discrimination based on essential and non-essential amino acid profiles, and the role of fish bioactive peptides in health and disease.

## Author contributions

SS and MM wrote the original draft of the article. LG and VD reviewed and edited the article. All authors contributed to the article and approved the submitted version.
